# Identifying Molecular Changes in Early Cervical Cancer Samples of Patients That Developed Metastasis

**DOI:** 10.3389/fonc.2021.715077

**Published:** 2022-01-11

**Authors:** Vera de Geus, Patricia C. Ewing-Graham, Willem de Koning, Maurits N. C. de Koning, Thierry P. P. van den Bosch, Alex L. Nigg, Casper H. J. van Eijck, Marta Jozwiak, Heleen J. van Beekhuizen, Dana A. M. Mustafa

**Affiliations:** ^1^ Department of Gynaecologic Oncology, Erasmus MC Cancer Institute, Rotterdam, Netherlands; ^2^ Department of Pathology, Tumor Immuno-Pathology Laboratory, Erasmus University Medical Center Rotterdam, Rotterdam, Netherlands; ^3^ Department of Pathology, Erasmus MC Cancer Institute, Rotterdam, Netherlands; ^4^ Department of Pathology, Clinical Bioinformatics Unit, Erasmus University Medical Center Rotterdam, Rotterdam, Netherlands; ^5^ Department of Research & Development Services, Delft Diagnostic Laboratory (DDL) Diagnostic Laboratory, Rijswijk, Netherlands; ^6^ Department of Surgery, Erasmus University Medical Center Rotterdam, Rotterdam, Netherlands

**Keywords:** early-stage cervical cancer, distant recurrence, cancer-related genes, immune microenvironment, local recurrence

## Abstract

Cervical cancer is one of the most common cancers in women worldwide. Patients diagnosed with early-stage cervical cancer have a good prognosis, however, 10-20% suffer from local or distant recurrent disease after primary treatment. Treatment options for recurrent cervical cancer are limited. Therefore, it is crucial to identify factors that can predict patients with an increased risk of recurrence to optimize treatment to prevent the recurrence of cervical cancer. We aimed to identify biomarkers in early-stage primary cervical cancer which recurred after surgery. Formalin-Fixed, Paraffin-Embedded surgical specimens of 34 patients with early-stage cervical cancer (FIGO 2009 stage 1B1) and 7 healthy controls were analyzed. Targeted gene expression profiling using the PanCancer IO 360 panel of NanoString Technology was performed. The findings were confirmed by performing immunohistochemistry stainings. Various genes, namely GLS, CD36, WNT5a, HRAS, DDB2, PIK3R2, and CDH2 were found to be differentially highly expressed in primary cervical cancer samples of patients who developed distant recurrence. In addition, The relative infiltration score of CD8+ T cells, CD80+CD86+ macrophages, CD163+MRC1+ macrophages, and FOXP3+IL2RA+ regulatory T cells were significantly higher in this group of samples. In contrast, no significant differences in gene expression and relative immune infiltration were found in samples of patients who developed local recurrence. The infiltration of CD8 and FOXP3 cells were validated by immunohistochemistry using all samples included in the study. We identified molecular alterations in primary cervical cancer samples from patients who developed recurrent disease. These findings can be utilized towards developing a molecular signature for the early detection of patients with a high risk to develop metastasis.

## Introduction

Worldwide there are approximately 700.000 new cases of cervical cancer, mostly affecting women of reproductive age (1). With 311.000 deaths annually, it is the fourth leading cause of cancer deaths in women (2). In 99.5% of the cases, cervical cancer is caused by the human papillomavirus (HPV) (3). The rise of effective primary and secondary prevention programs has substantially reduced the number of new cases in high-income countries (4). However, despite falling incidence and mortality rates in high-income countries, those patients diagnosed with recurrent cervical cancer still have few curative treatment options and their median survival is 10-12 months (5).

Early cervical cancer (FIGO 2009 stage I-IIA) is treated by either primary radiotherapy or a radical hysterectomy (6). Adjuvant radiotherapy is indicated after surgery when one of the following risk factors for recurrence is present: lymphovascular space invasion (LVSI), tumor size >4 cm, invasion depth >2/3 or >15 mm. In the case of positive lymph nodes (LNs) and/or parametrial invasion and/or positive margins, patients are offered adjuvant chemoradiation. Early cervical cancer is considered to have a low risk for recurrence, but 10-20% of patients will still suffer from a recurrence (7, 8). In advanced (high-risk) cervical cancers (FIGO 2009 stage IIB-IV) 70% of patients experience a recurrence, despite multimodal therapy with chemotherapy, radiotherapy, and/or hyperthermia (6). The division into low- and high-risk seems irrelevant to patients who are classified as low-risk but suffer from recurrence. It is crucial to try and identify factors that can help classify patients according to the risk of recurrence, so allowing optimal preventative treatment.

To date, the subtyping of cervical cancer is based solely on histology, there is no molecular subtyping. Previous research has shown that the genes SHKBP1, ERBB3, CASP8, HLA-A, TGFBR2, PIK3CA, EP300, FBXW, HLA-B, PTEN, NFE2L2, ARID1A, KRAS, and MAPK1 are frequently mutated in cervical cancer (9). The latest research on molecular subgroups in cervical cancer has identified three mRNA-based subgroups: high expression of keratin gene family members, low expression of keratin gene family members, and adenocarcinoma-rich cluster (9). In the keratin high cluster, no KRAS mutation was observed. In the adenocarcinoma-rich cluster, no mutations in the HLA-A gene were identified. Additionally, a subgroup of endometrial-like cervical cancers was identified. This group contained high frequencies of KRAS, ARID1A, and PTEN mutations (9). No connection with clinical outcomes has as yet been found based on these groupings.

Cervical cancer is in most cases a human papillomavirus (HPV) related disease, and the immune system plays an important role in the development of these tumors (10). Due to an impaired immune response HPV infection persists resulting in the expression of viral oncoproteins E6 and E7. These oncoproteins interact with tumor suppressor genes p53 and retinoblastoma (pRb) leading to genomic instability, which in turn could lead to malignant transformation of the cervix epithelial cells (11). The impaired immune response is based on defective local cellular immunity. HPV suppresses inflammatory signaling in the epithelium leading to low epithelial cytokine and chemokine production. This prevents dendritic cell recruitment and allows viral immune escape and persistence (10). The viral infection causes various changes in the immune microenvironment of cervical cancer. Investigating these changes may lead to discovering new therapeutic options to treat cervical cancer patients.

The ultimate aim of this work is to identify biomarkers in primary cervical cancer tissue to predict tumor recurrence after surgery. Therefore, we investigated the differences at the molecular level (genes, immune cell types, and pathways) in the primary cervical cancer samples of patients who suffer from local- or distant recurrence, also comparing these with tissue from patients who had no recurrence. The results of this pilot study are the first step towards a molecular classification in cervical cancer that might help develop patient stratification and may give more insight into the progression of this deadly disease.

## Materials And Methods

### Patient Samples Collection

Tissue samples and clinical information from cervical cancer patients were selected retrospectively. All patients diagnosed with squamous cell carcinoma or adenocarcinoma of the uterine cervix who were treated with a radical hysterectomy in the Erasmus Medical Center Rotterdam from 1995 to 2017 were screened for inclusion. Samples were included if they met the following criteria: FIGO 2009 1B1 cervical cancer, primary treatment radical hysterectomy, and development of local or distant recurrent disease after primary treatment. In addition, samples of patients who did not develop recurrence were included in the study as a control group. The retrospective database was also screened for patients who underwent a hysterectomy for uterus myomatosus without cervical dysplasia to collect normal cervical tissue. Patient samples were divided into four groups: cervical cancer without recurrence (CCNR), cervical cancer that eventually developed local recurrence (CCLR), cervical cancer that eventually developed distant recurrence (CCDR), and healthy controls (HC). The follow-up time was at least five years for the cervical cancer groups. The slides and formalin-fixed, paraffin-embedded (FFPE) tissue blocks of the cases were retrieved from the departmental archive. Hematoxylin-eosin (HE) stained slides of all cases studied were reviewed for pathologic diagnosis by a pathologist experienced in gynaecopathology. Areas enriched with tumor tissue and/or normal cervical epithelium were marked for each case. The study was approved by the medical-ethical committee of Erasmus Medical Center (MEC-2019-0793). The samples were used only if the patients signed the informed consent.

### RNA Isolation

RNA was isolated using the RNeasy^®^ FFPE Kit (Qiagen, Hilden, Germany) according to the manufacturer’s directions. RNA concentrations and quality were measured using the Agilent 2100 BioAnalyzer (Santa Clara, CA, USA). To correct for degradation of the RNA, the percentage of fragments of 300–4000 nucleotides was used to calculate the corrected concentrations. For each sample, 300 ng of total RNA, with a maximum of 7 μL (>42.8 ng/μL), was used.

### Targeted Gene Expression Analysis

Targeted gene expression analysis was performed using the PanCancer IO 360™ Panel and the nCounter^®^ FLEX system (NanoString Technologies Inc., Seattle, USA). The panel is composed of 750 genes involved in the interplay between tumor, microenvironment, and immune response in cancer and 20 housekeeping genes. Hybridization was performed at 65°C for 17 hours using a SimpliAmp Thermal Cycler (Applied Biosystems, Foster City, CA, USA). Gene counting was performed by scanning 490 Fields of View (FOV).

### Statistical Analysis

Data were analyzed using the nSolver software (version 4.0) and the advanced analysis model (version 2.0, NanoString, Seattle, USA). Raw gene counts were normalized using the most stable housekeeping genes that were included in the panel, selected by the geNorm algorithm (12). Negative controls were used to determine the background threshold based on calculating the average count of the 8 negative controls +2 standard deviations. Genes that had counts above the threshold were considered as detected genes. The normalized data were used to calculate differentially expressed (DE) genes (p-value < 0.05), score the relative abundance of immune cell types, and calculate variations in the pathways. To determine the DE genes the DE Algorithm which was incorporated in the software was applied to the data. Subsequently, the Benjamini-Hochberg procedure was performed to correct for multiple testing. Disease-free survival was determined as the time to recurrence in months from the last day of treatment.

### Cell Type Calculation

Data of all gene expression (not only the differentially expressed genes) were used to estimate the relative infiltration of immune cells following the method developed within the nSolver Advanced Analysis module by Danaher et al. (13). The immune cell definition method using marker genes was improved to increase the specificity by “personalizing” the gene markers used to identify immune cell types for each type of tissue (e.g., cervical cancer) (14). In brief, the gene markers for each immune cell type were selected based on literature, then the correlation of genes that mark one cell type was calculated. Genes that showed a high correlation in all samples (R2 > 0.6, and slope close to 45 degrees), but did not correlate with any other genes were considered as marker genes that were used to identify a specific type of immune cells. The advanced analysis module of nSolver software enables calculating relative scores for immune cells and cancer-related pathways (15). The significance of the relative abundance of cell types between the group of samples was calculated using the two-sided Student’s t-tests, with p-value <0.05 as a cut-off for significant findings. Statistical analyses were performed with IBM SPSS Statistics (version 25.0, IBM corporation, Armonk, USA).

### HPV Genotyping

In collaboration with DDL Diagnostic Laboratory, Rijswijk, HPV genotyping was performed. Proteinase K digestion was done on two sections of 8 μm per sample. The total DNA from all the samples was first tested by quantitative PCR targeting the human RNaseP open reading frame to confirm the presence of amplifiable human DNA. HPV SPF10-LiPA25 version 1 (Labo Bio-medical Products, Rijswijk, the Netherlands) was used to detect the HPV genotype in all samples. This short-PCR-fragment assay amplified a 65-bp fragment of the L1 open reading frame of HPV genotypes. DNA specimens were tested for the presence of HPV DNA by PCR amplification using the SPF10 primer system. Ten μL of every PCR reaction was tested on the presence of HPV SPF10 PCR products using the SPF10 DNA enzyme immunoassay (DEIA) detection system. The DEIA can detect DNA from at least 67 HPV types. Line probe assay (LiPA25) was then used to analyze the samples found positive or borderline for HPV by DEIA (OD450 (optical density at 450 nm) > 0.160) by reverse hybridization with type-specific probes for 25 high-risk HPV and low-risk types: HPV 6, 11, 16, 18, 31, 33, 34, 35, 39, 40, 42, 43, 44, 45, 51, 52, 53, 54, 56, 58, 59, 66, 68/73, 70, and 74 (16).

### Immunohistochemistry

Sections of 4 µm thickness of all samples included in the study were mounted to a coated slide. Automated immunohistochemistry was performed using the Ventana Benchmark ULTRA (Ventana Medical Systems Inc., Oro Valley, USA). Sequential sections were stained for CD8 (Ventana, SP57 Y04591) and FOXP3 (Thermofisher, 236A/E7 4339062). In brief, following deparaffinization and heat-induced antigen retrieval, the tissue samples were incubated according to their optimized time and protocol with the antibody of interest (**Supplementary Table 1**). Incubation was followed by haematoxylin II counterstain (#790-2208) for 8 minutes and then a bluing reagent (#760-2037) for 8 minutes according to the manufacturer’s instructions (Ventana Medical Systems Inc., Oro Valley, USA). Positive control of tonsils tissue samples was added to every slide and stained together with the cervical cancer tissue. In addition, tonsils tissue samples were stained with CD8 and FOXP3 (positive control) and in a separate slide were treated with PBS (negative control) (**Supplementary Figure 1**).

Histology scoring was performed using a designed algorithm in ImageJ software. The algorithm is based on calculating the surface of the positive staining relative to the total surface area. The stained slides were scanned by using the NDP slide scanner (Hamamatsu Nanozoomer 2.0HT, Hamamatsu City, Japan) at 40X. From every slide, three photos were exported resulting in images of 7680X4320 pixels with a pixel size of 228 nm (5x magnification on a screen, exported as 20x magnification by using the NDP Viewer). To scour the slides digitally, a region of interest (ROI) was drawn, and the surface of all positively stained cells was measured in pixels and compared to the total surface in pixels of cells. This resulted in quantitative ratios that were used in the data analysis.

## Results

### Clinical Characteristics

**Figure 1:**
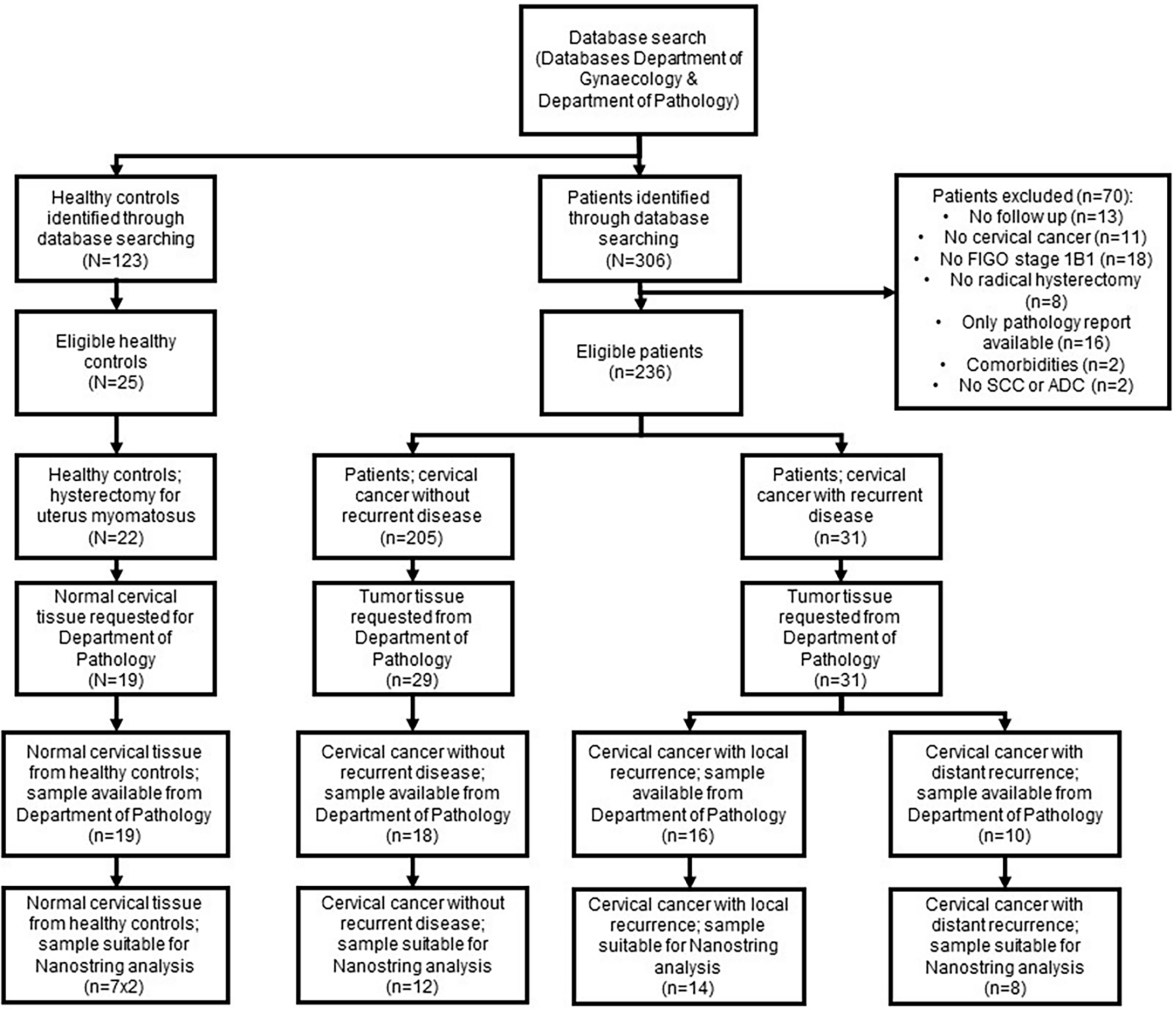
Flowchart patient samples. Flowchart demonstrating the number of patients samples included in the study and the excluded samples with the reasons.

Between 1995 to 2017, 250 patients were treated for cervical cancer FIGO 2009 stage 1B1 with a radical hysterectomy at the Erasmus MC Cancer Institute; 31 patients (12.4%) developed recurrent disease after primary surgical treatment. Tissue samples of 22 patients were available and suitable to be used in this study (**Figure 1**). The 22 samples from patients who developed recurrence were divided into local recurrent (CCLR, n=14) and distant recurrent disease (CCDR, n=8). To ensure that the findings were recurrence-related, twelve patients surgically treated for cervical cancer stage 1B1 without recurrence (CCNR) were included. Healthy cervical tissue from seven patients treated for a benign gynecologic disorder (HC) were used as controls. Clinicopathological characteristics of all patients are depicted in **Table 1**.

**Table 1:** Clinicopathological characteristics.

	CCLR (n = 14)	CCDR (n = 8)	CCNR (n = 12)	*p*-value^a^
Age	45.6 [32 – 69]	47.6 [37 – 60]	42.4 [32 – 61]	0.51
Histology				0.12
Squamous cell carcinoma	8 (57.1)	7 (87.5)	5 (41.7)	
Adenocarcinoma	6 (42.9)	1 (12.5)	7 (68.3)	
Surgical approach				**<0.01**
Laparotomy	14 (100.0)	8 (100.0)	3 (25.0)	
Robot-assisted	0 (0.0)	0 (0.0)	9 (75.0)	
LVSI				0.11
Yes	6 (42.9)	7 (87.5)	6 (50.0)	
No	8 (57.1)	1 (12.5)	6 (50.0)	
TNM stage, post-operative				**<0.01**
T1B1 N0 M0	14 (100)	2 (25.0)	12 (100)	
T1B1 N1 M0	0 (0)	6 (75.0)	0 (0)	
Adjuvant therapy				**<0.01**
Not indicated	12 (85.7)	0 (0.0)	10 (83.3)	
Radiotherapy	2 (14.3)	6 (75.0)	2 (17.7)	
Chemoradiation	0 (0.0)	2 (25.0)	0 (0.0)	
HPV subtype				
HPV-16	9* (69.2)	5 (62.5)	8 (66.7)	
HPV-18	2 (15.4)	2 (25.0)	3 (25.0)	
Other high-risk HPV	–	1 (12.5)	1 (8.3)	
No HPV	2 (15.4)	–	–	
Time to recurrence, months	17 [7-139]	39 [10-90]		0.48
Disease status last follow-up				**<0.01**
Alive, no evidence of disease	9 (64.3)	3 (37.5)	12 (100)	
Dead with disease	5 (36.7)	5 (62.5)	0 (0)	

Values are presented as numbers (%) or mean [range]. The numbers in bold are statistically significant. CCLR, Cervical cancer local recurrence; CCDR, Cervical cancer distant recurrence; CCNR, Cervical cancer no recurrence; FIGO, International Federation of Gynaecology and Obstetrics; LVSI, lymphovascular space invasion; TNM, Tumour Node Metastasis Classification of Malignant tumors. a Represents the P value between CCLR vs. CCDR vs. CCNR and the value in bold indicates a significant difference between the groups (p<0.05). *One patient was infected with both HPV-16 and HPV-18.

^a^Represents the p-value between CCLR vs. CCDR vs. CCNR and the value in bold indicates a significant difference between the groups (p < 0.05).

### Up-Regulation of Cancer-Related Genes in Primary Cervical Cancer Samples of Patients Who Developed Distant Metastasis

**Figure 2:**
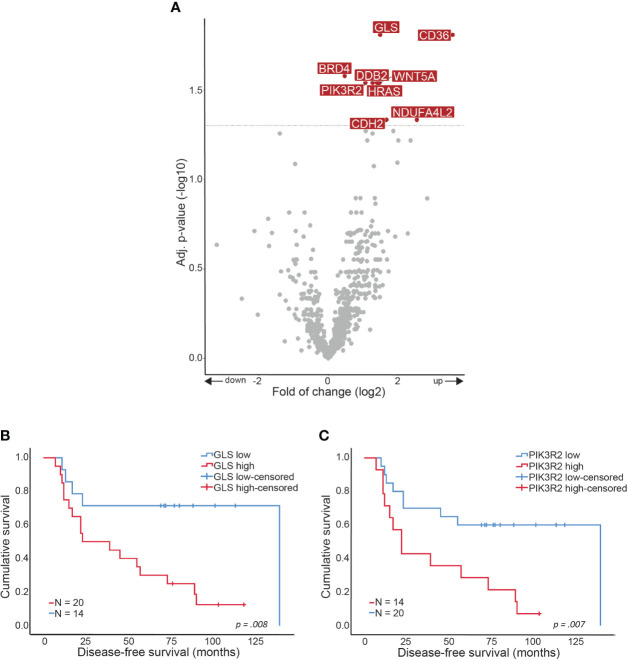
Gene expression alterations in CCDR. Volcano plot showing over- and underexpressed genes in CCDR. Each dot represents a gene of the nCounter^®^ PanCancer IO 360 Gene Expression Panel. Y-axis: p-values after the Benjamini-Hochberg procedure (-log10 scale). X-axis: Fold of change is calculated with CCNR as baseline reference (log2 ratio). Arrows indicate up- or downregulation. Kaplan-Meier curves showing disease-free survival in months related to gene expression. **(A)** CCDR vs. CCNR **(B)** GLS gene expression and **(C)** PIK3R2 gene expression. CCNR, Cervical cancer no recurrence; CCDR, Cervical cancer distant recurrence.

Comparing CCDR to CCNR resulted in discovering 7 DE genes (p-value <0.05), namely GLS, CD36, WNT5a, HRAS, DDB2, PIK3R2, and CDH2 that were all up-regulated in the CCDR group (**Figure 2A**). High fold-of-change (FOC = 11.9) was measured for CD36, however, this was a result of one outlier sample. Correlating the expression of the DE genes to the clinical data highlighted the significant association of GLS (p=0.008) and PIK3R2 (p=0.007) genes with shorter disease-free survival (DFS) (**Figures 2B, C**).

### Primary Cervical Tumor Tissue From CCLR and CCNR Are Similar

The comparison of CCLR and CCNR resulted in no DE genes between the two groups of samples (**Supplementary Figure 2**). The analysis showed that some genes were significant at a p-value < 0.05, however, this significance did not hold after multiple corrections.

### Not All Cancer-Related Genes Were Differentially Expressed in Cervical Cancer Samples

**Figure 3:**
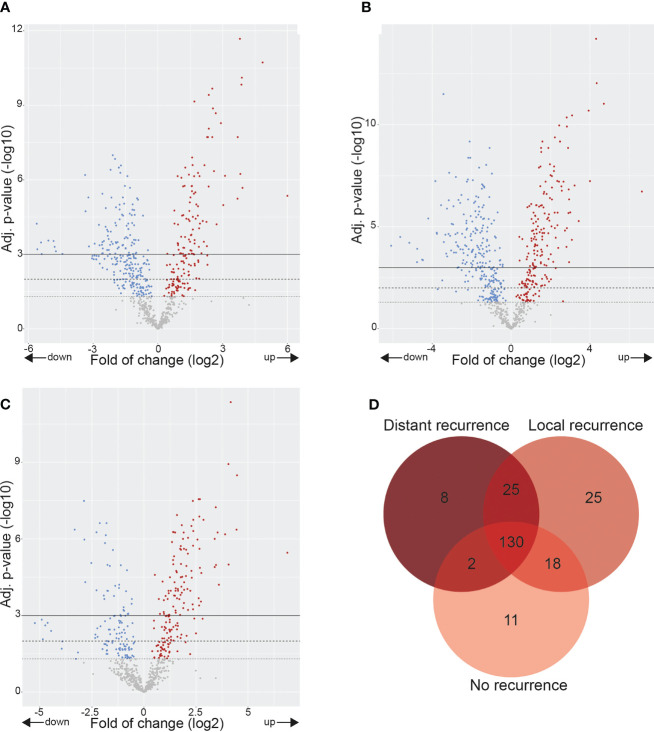
Gene expression alterations in cervix tumors. Volcano plots showing over- and underexpressed genes in cervix tumors. Each dot represents a gene of the nCounter^®^ PanCancer IO 360 Gene Expression Panel. Y-axis: p-values after the Benjamini-Hochberg procedure (-log10 scale). X-axis: Fold of change is calculated with healthy controls as baseline reference (log2 ratio). **(A)** CCNR vs. healthy controls. **(B)** CCLR vs. healthy controls. **(C)** CCDR vs. healthy controls. **(D)** Venn diagram showing differentially overexpressed genes in CCNR, CCLR, CCNR relative to healthy controls. CCNR, Cervical cancer no recurrence; CCLR, Cervical cancer local recurrence; CCDR, Cervical cancer distant recurrence.

Comparing HC to the three cervical cancer groups showed that many cancer-related genes were differentially expressed (**Figures 3A** **–C**). Only 130 of the 750 examined DE genes overlapped between HC and CCNR, CCLR, and CCDR, and only a few genes were found to be specifically upregulated in each of the cervical cancer groups (**Figure 3D**). These results highlight that the molecular makeup of the cervical cancer samples was different from healthy controls.

### The Highest Variation of Immune Cell Infiltration Was Presented in CCDR

**Figure 4:**
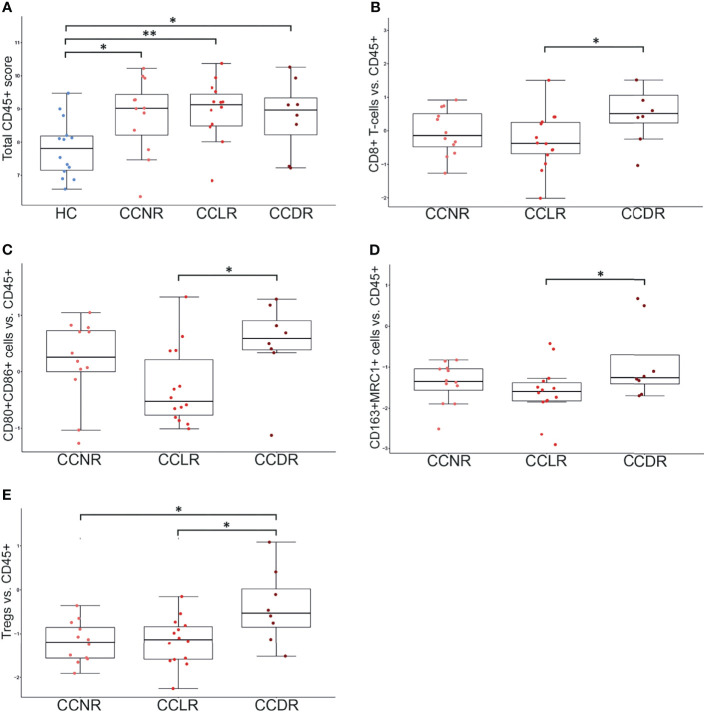
Immune cell populations in cervical cancer tissue. Each dot represents one sample. Y-axis indicates the immune cell score (ratio of given cell type relative to CD45+ immune cells). *P < 0.05, **P < 0.01. **(A)** Total CD45+ lymphocyte score. HC show significantly lower CD45+ cells compared to any cervical cancer group. **(B)** CD8+ T cells vs. CD45: significant differences between HC vs. each cervical cancer group and CCLR vs. CCDR. **(C)** CD80+CD86+ Macrophages vs. CD45: significant differences between HC vs. each cervical cancer group and CCLR vs. CCDR **(D)** CD163+MRC1+ Macrophages vs. CD45: significant differences between HC vs. each cervical cancer group and CCLR vs. CCDR. **(E)** Tregs vs. CD45: significant differences between HC vs. CCLR, HC vs. CCNR, CCNR vs. CCDR, and CCLR vs. CCDR. Tregs, Regulatory T cells; HC, Healthy controls; CCLR, Cervical cancer local recurrence; CCDR, Cervical cancer distant recurrence; CCNR, Cervical cancer no recurrence.

**Figure 5:**
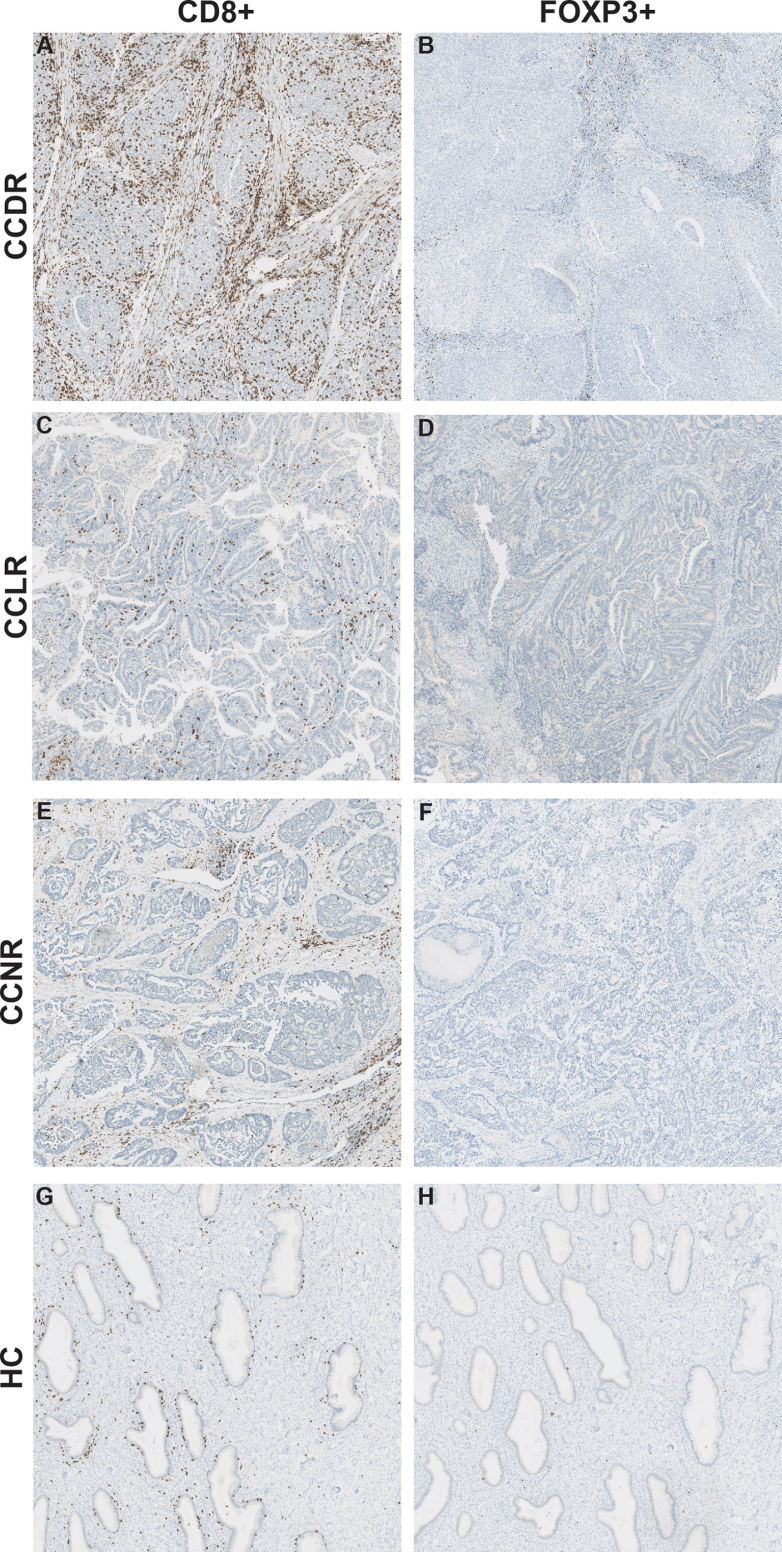
CD8+ cells and FOXP3+ cells in cervical cancer tissue. Representative images for immunohistochemical staining of CD8 and FOXP3 in cervical cancer tissue. The CD8 and FOXP3 images are from the same patient for each cervical cancer group. **(A)** CD8+ cells in CCDR. **(B)** FOXP3+ cells in CCDR. **(C)** CD8+ cells in CCLR. **(D)** FOXP3+ cells in CCLR. **(E)** CD8+ cells in CCNR. **(F)** FOXP3+ cells in CCNR. **(G)** CD8+ cells in HC. **(H)** FOXP3+ cells in HC.

**Figure 6:**
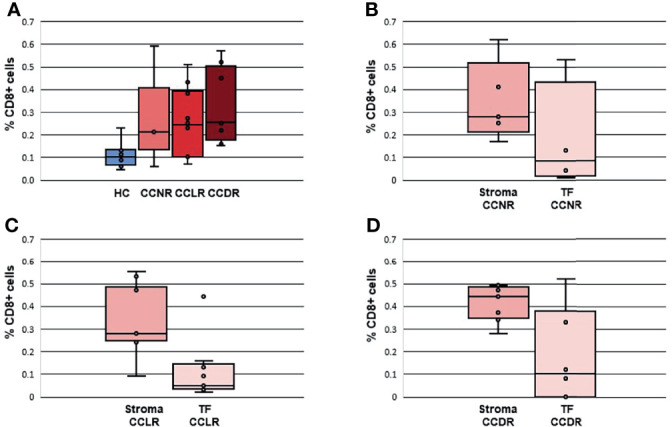
Density of CD8+ cells in cervical cancer tissues and healthy controls. The percentage of CD8+ cells is calculated according to surface CD8+ cells/surface of all cells *100. **(A)** Density CD8+ cells in all groups. **(B)** Density CD8+ cells in CCNR comparing stromal tissue and tumor fields. **(C)** Density CD8+ cells in CCLR comparing stromal tissue and tumor fields. **(D)** Density CD8+ cells in CCDR comparing stromal tissue and tumor fields. TF, Tumor fields; HC, Healthy controls; CCLR, Cervical cancer local recurrence; CCDR, Cervical cancer distant recurrence; CCNR, Cervical cancer no recurrence.

Gene expression results were utilized to estimate the relative scoring of immune cells. Marker genes that were used to define immune cells and their QC results are summarized in **Supplementary Table 2**. The relative scores of total infiltrating lymphocytes (TILs) were calculated between the groups based on the expression of CD45 (PTPRC) and were found to be higher in all cervical cancer groups compared to HC (**Figure 4A**). However, the total infiltration of CD45+ TILs (PTPRC) was not found to be significantly different between the sub-groups of cervical cancer (**Figure 4A**). Importantly, the composition of immune cells that infiltrated the cervical cancer tissue (relative to the total TILs) was found to be significantly different. Various immune cells type scores (relative to total TILs) were found to be significantly higher in CCLR compared to CCDR, namely: CD8+ T cells (p=0.037), CD80+CD86+ macrophages (p=0.046), CD163+ MRC1+ macrophages (p=0.023), and FOXP3+ IL2RA+ regulatory T cells (Tregs, p=0.015) (**Figures 4B** **–E**). Tregs were also statistically significantly different between CCDR vs. CCNR (p=0.013). The infiltration of CD8+ cells in CCDR samples was measured by IHC. An example of the immunohistochemistry results is shown in (**Figures 5A, C, E, G**). The statistical analysis of immunohistochemistry for all the samples showed that the CD8+ density was significantly higher in the cervical cancer groups compared to healthy controls (**Figure 6A**). Comparing CD8+ density in the tumor fields to the stromal tissue in CCNR did not show a significant difference (**Figure 6B**). Nevertheless, significantly higher CD8+ density in the stromal tissue in both CCLR (p=0.004) and CCDR (p=0.01) was observed (**Figures 6C, D**). Although the total CD8+ density showed no significant differences between CCLR and CCDR, more samples of CCDR showed high CD8+ density in the tumor fields.

### The Density of FOXP3+ Cells Is Similar in Healthy Controls Compared to Cervical Cancer Tissue Samples

To validate the result of a significantly higher relative infiltration of Tregs in CCDR, IHC of FOXP3 was performed. No significant differences were observed between healthy controls and the cervical cancer groups (**Figures 5B, D, F, H**, **Supplementary Figure 3**). The expression of FOXP3 and IL2RA, the marker genes to calculate the relative infiltration of Tregs, were both evenly expressed in healthy cervical cancer tissue and cervical cancer tissues (**Supplementary Figure 4**). Therefore, we concluded that both genes FOXP3 (transcriptional factor) and IL2RA (cytokine) have an important function in healthy cervical tissue samples and cannot be used as accurate marker genes to identify Tregs cells in cervical cancer.

## Discussion

Cervical cancer FIGO 2009 stage 1B1 is generally treated with a radical hysterectomy and pelvic lymph node dissection. Despite this extensive surgery, the disease recurs in 10-20% of cases (5, 7, 8). To date, no genetic markers are known to predict whether cervical cancer will recur after surgery. To identify such markers, we performed a gene expression analysis on primary tumors from 34 cervical cancer patients with or without recurrent disease after surgery. We used normal cervical tissue samples obtained from 7 patients treated with hysterectomy for uterus myomatosus as normal controls. In addition to analyzing the DE genes, we investigated the role of the immune cells in our cohort.

NanoString PanCancer IO 360™ expression analysis showed some genetic alterations in CCDR vs. CCNR. Namely, GLS, CD36, WNT5a, HRAS, DDB2, PIK3R2, and CDH2 were significantly DE between cervical cancer without recurrence and cervical cancer with distant recurrence. High GLS and high PIK3R2 expression were also associated with a shorter DFS. Therefore, these two genes are most interesting in the light of developing a predictive biomarker of disease recurrence. GLS is an enzyme involved in cellular metabolism which generates glutamate from glutamine. Dysregulation of glutamine metabolism results in the proliferation of cancer cells (17). Saha et al. performed a systematic multi-omics analysis to test GLS as a prognostic biomarker (18). They found that GLS was overexpressed in breast, esophagus, head-and-neck, and hematologic cancers and was associated with poor prognosis. In our cohort, high GLS expression is also associated with a shorter DFS. However, we acknowledge that drawing hard conclusions of DFS is hard considering the low number of samples included in our study. In a previous study, GLS was shown to be highly expressed in the kidney, brain, intestine, and lymphocytes, but not in the cervix (19). To exclude a contribution of GLS expression from lymphocytes infiltrated in the cervical tissue samples, the CD45 score in our cohort of samples was not found to be higher in the CCDR group. Therefore, we think that the high expression of GLS in CCDR samples is probably due to the increased metabolic activity of metastatic cancer cells. However, we did not determine the cell of origin of GLS expression in the current study.

Dysregulation of the PI3K/PTEN pathway is a common event in cancer. PIK3R2 encodes for the p85β regulatory subunit of PI3K. Previous studies showed that the expression of PIK3R2 increases with the advanced tumor stage in melanoma, breast, and squamous cell lung carcinoma (20–22). Also, overexpression of PIK3R2 induced metastasis in mouse model studies (21). In cervical cancer, it has been shown that PI3K is amplified and activated in HPV-induced cervical cancer (23). However, an oncogenic role for p85β is not yet discovered in cervical cancer. Our study showed that PIK3R2 expression is also upregulated in cervical cancer which eventually proceeds to distant recurrent disease. Measuring the expression of GLS and PIK3R2 in the resected cervical cancer samples may be a future step in the identification of patients with a high risk of recurrence. Therapies targeting PIK3R2 are currently investigated but the results are disappointing so far (24). However, the effect of depletion of PIK3R2 is greater in solid tumors than in cultured cells, hinting that tumor microenvironment may play a role in gene expression.

We tried to validate our results using TCGA data. We found RNA sequencing data of 87 of stage 1b1 cervical cancer, 70 of them received radical hysterectomy as an initial treatment. The number of samples of local and distant recurrence was very limited. Only 4 samples were of patients who developed local recurrence, and 7 of distant recurrence. While 41 samples of patients who did not develop recurrence were found (**Supplementary Figure 5**). Therefore, we could not validate the discovered differentially expressed genes using the TCGA data. It is difficult to compare data of 41 samples to that of 4 or 7 samples. The low number of samples we found in the TCGA data highlights the importance of our study. While we acknowledge that the number of samples included in our study is relatively low, they are comparable to the number of samples available in the TCGA data.

We found a significantly higher CD8+/CD45+ ratio in CCDR compared to CCLR. Interestingly, no significant difference was found between CCDR and CCNR. In cervical cancer, Tregs are recognized as suppressors of the effector T cells (25). In our study, Tregs/CD45+ ratios were significantly higher in CCDR compared to CCNR and CCLR. Our IHC analysis validated the presence of CD8+ T cells and Tregs in cervical tumors. However, it was hard to validate our results for the relative immune infiltration based on the marker genes. The immunohistochemistry assay we performed to validate this result showed no clear difference in the number of CD8+ T cells and Tregs comparing CCDR to CCLR. However, IHC analysis did show that CD8+ T cells and Tregs density were lower in the tumor tissue itself than in peritumoral tissue.

We found similar mRNA levels for FOXP3 in normal cervical tissue as in cervical cancer tissue. FOXP3 is a specific marker for Tregs and functions as a master regulator in the development and function of Tregs (26). In cervical cancer tissue, it is known that FOXP3 expression is upregulated, however, little is known about FOXP3 expression in normal cervical tissue. Luo et al. described no expression or weak expression of FOXP3 in normal cervical tissues detected by Western blot assays (27). The human protein atlas reports low expression of FOXP3 in normal cervical tissues (28). Our results suggest that FOXP3 has a different function in normal cervical tissue than in cervical cancer tissue. We postulate that in normal cervical tissues the mRNA of FOXP3 is not translated to a protein. In addition, FOXP3 can act as a transcriptional factor that promotes cell growth occurs continuously in the cervix.

Our gene expression analysis showed no significant genetic alterations in CCLR vs. CCNR, which could be explained by the fact that both groups are similar. During surgery, the uterus including the cervix and adjacent tissue is removed, and the surgical margins are later checked by a pathologist. In CCLR cases, it may be that despite the margin appearing free there remain tumor cells in the patient which grow and become apparent as a recurrent disease in the original location of the cervix. Because these tumor cells originate directly from the primary tumor they have the same genetic profile and, no significant DE genes are observed between samples of cervical cancer without recurrence and samples of cervical cancer with local recurrence. Adequate pathology protocols for examining the margins of the specimen are therefore an important point.

In conclusion, we identified molecular alterations in primary cervical cancer samples of patients that developed distant recurrence (metastasis). These findings can be utilized towards developing a molecular signature for the early detection of patients with a high risk to develop metastasis.

## Data Availability Statement

The data presented in the study are deposited in the Gene Expression Omnibus (GEO) repository, accession number GSE192897.

## Ethics Statement

The study involved human samples that were reviewed and approved as a non-WMO study by the medical-ethical committee of Erasmus Medical Center (MEC-2019-0793). The study was performed on leftover tissue of surgical specimens. The Dutch national guidelines state that no ethical approval is required for the use of anonymous leftover tissue (www.federa.org) and this is also part of a standard treatment agreement with patients at Erasmus MC.

## Author Contributions

VG, MK, TB, and DM: experiments. VG, WK, and DM: analysis. PE-G: pathology revision. CE, MJ, HB, and DM: Concept. AN: software development. VG and DM: writing. All authors reviewed and corrected the manuscript. All authors contributed to the article and approved the submitted version.

## Funding

This work was supported by generous contributions to the Erasmus Trustfonds (Hoijerik fund) and by the Support Casper Foundation (www.supportcasper.org).

## Conflict of Interest

The authors declare that the research was conducted in the absence of any commercial or financial relationships that could be construed as a potential conflict of interest.

## Publisher’s Note

All claims expressed in this article are solely those of the authors and do not necessarily represent those of their affiliated organizations, or those of the publisher, the editors and the reviewers. Any product that may be evaluated in this article, or claim that may be made by its manufacturer, is not guaranteed or endorsed by the publisher.
