# Supplementary material for: Identifying Molecular Changes in Early Cervical Cancer Samples of Patients That Developed Metastasis
Source: Front Oncol. 2022 Jan 11;11:715077. doi: 10.3389/fonc.2021.715077 (PMC8787153; doi:10.3389/fonc.2021.715077)
Supplement: Supplementary file 1 [file DataSheet_1.pdf]

Supplementary Material

1 Supplementary Tables

Supplementary Table 1 Immunohistochemistry information

**Antibody**	**CD8**	**FOXP3**
**Type**	Anti-Rabbit	Anti-Mouse
**Concentration**	0.35 µg/ml	0.5 µg/ml
**Company**	Ventana	Thermofisher
**Clone**	SP57	236A/E7
**Lot number**	Y04591	4339062
**Procedure**	Optiview CC1 16’	Optiview CC1 32’
**Antibody incubation time**	32 minutes	32 minutes

Supplementary Table 2 Cell type-specific marker genes for immune cells

Cell name	Marker genes	CCNR		CCLR		CCDR		HC	
		C	Slope	C	Slope	C	Slope	C	Slope
**CD80⁺/CD86⁺ Macrophages**	*CD80/CD86*	0.85	0.81	0.78	1.12	0.96	0.77	0.88	0.71
**CD163⁺/MRC1⁺ Macrophages**	*CD163/MRC1*	0.65	0.84	0.66	1.06	0.83	0.61	0.52	0.64
**Plasma B-cells**	*CD27/CD38*	0.82	0.87	0.69	0.92	0.61	0.67	0.28	0.32
	CD27/SLAMF7	0.90	0.94	0.95	0.86	0.94	1.14	0.92	0.66
	SLAMF7/CD38	0.91	0.88	0.70	0.47	0.71	0.78	0.23	0.14
**CD4⁺ T-cells**	*CD4/SELL*	0.79	0.97	0.63	0.92	0.84	1.35	0.61	1.34
**Neutrophils**	*CSF3R/FPR1*	0.77	0.66	0.91	0.84	0.95	0.93	0.77	0.68
	FPR1/FCGR3a/b	0.77	0.43	0.84	0.69	0.89	0.39	0.88	1.06
	CSF3R/ FCGR3a/b	0.51	0.76	0.81	0.79	0.82	0.37	0.76	1.03
**Monocytes**	*TLR2/CD14*	0.69	0.73	0.83	0.68	0.61	0.53	0.18	0.38
	TLR2/TLR4	0.72	0.67	0.83	0.76	0.71	0.78	0.39	0.62
	TLR4/CD14	0.88	0.77	0.88	0.97	0.84	1.06	0.39	0.3
**Tregs**	*FoxP3a/IL2RA*	0.73	0.71	0.75	0.62	0.90	0.80	0.95	0.92
**CD45⁺ lymphocytes**	*CD45RO/PTPRC*	0.95	1.04	0.98	0.88	0.99	1.04	0.97	1
**CD8⁺ T-cells**	*CD8A/CD8B*	0.68	0.62	0.73	0.74	0.62	0.63	-0.14	-0.13
**NK cells**	*XCL1/2/NRC1*	0.72	1.12	0.32	0.38	0.84	0.77	0.89	1.57
**T-cells**	*CD6/CD3G*	0.80	0.91	0.57	0.62	0.92	0.90	0.94	0.96
	CD6/SH2D1A	0.84	1.24	0.82	0.83	0.97	1.09	0.97	1.12
	CD6/TRAT1	0.77	1.03	0.86	0.84	0.90	0.84	0.91	0.74
	CD6/CD3E	0.89	1.03	0.70	0.72	0.94	0.69	0.71	0.69
	CD3G/SH2D1A	0.92	1.18	0.87	0.81	0.96	1.09	0.92	1.03
	CD3G/TRAT1	0.91	1.07	0.79	0.71	0.94	0.90	0.98	0.79
	CD3G/CD3E	0.93	0.95	0.91	0.86	0.94	0.69	0.67	0.64
	SH2D1A/TRAT1	0.96	0.88	0.87	0.84	0.96	0.81	0.88	0.63
	SH2D1A/CD3E	0.95	0.74	0.90	0.91	0.94	0.62	0.88	0.61
	TRAT1/CD3E	0.95	0.82	0.78	0.82	0.91	0.70	0.64	0.76

2 Supplementary Figures

Immunohistochemistry positive and negative controls

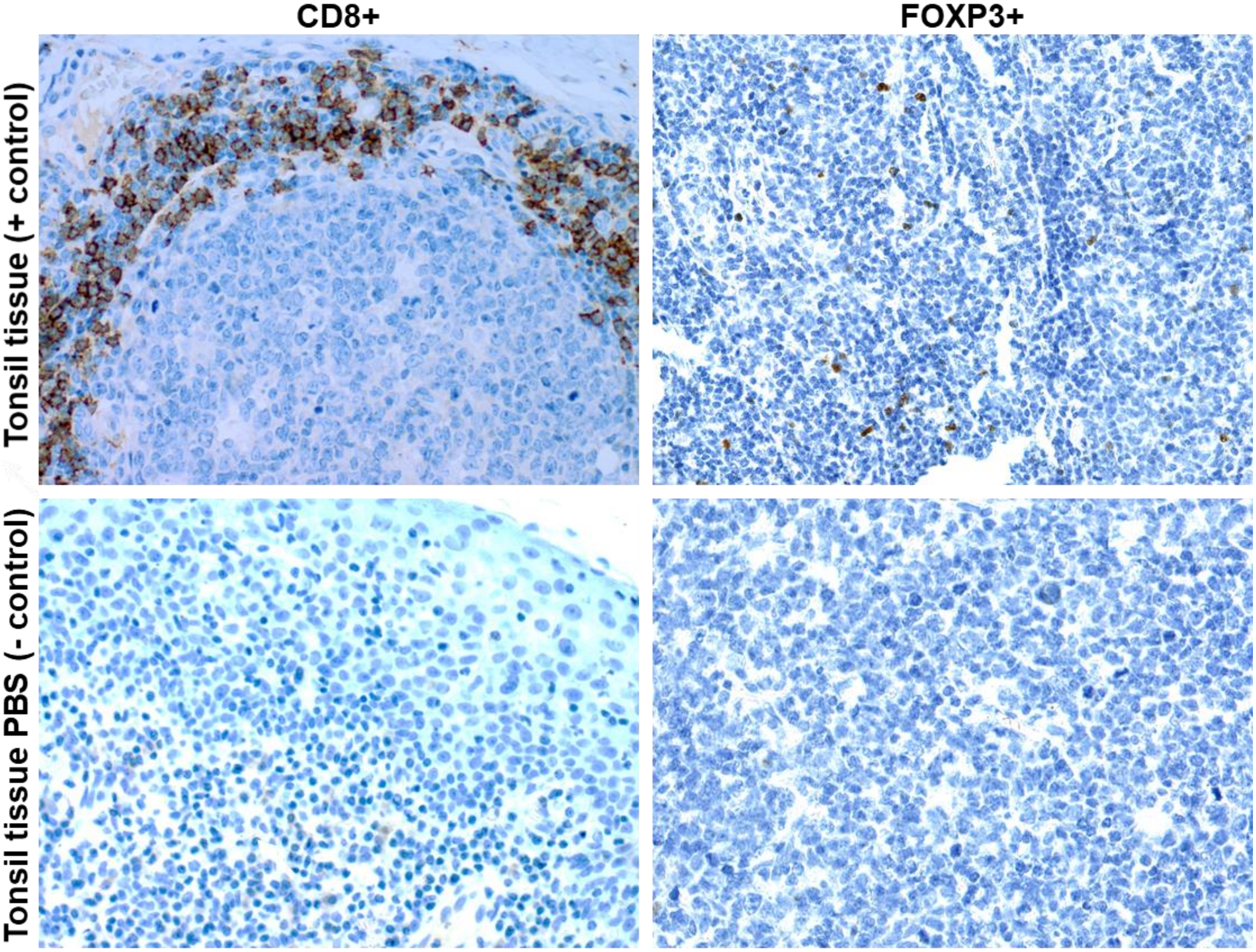

Supplementary Figure 1. Representative images for immunohistochemical staining of CD8 and FOXP3 in tonsil tissue (positive control) and tonsil tissue treated with PBS (negative control).

Gene expression alterations in CCLR

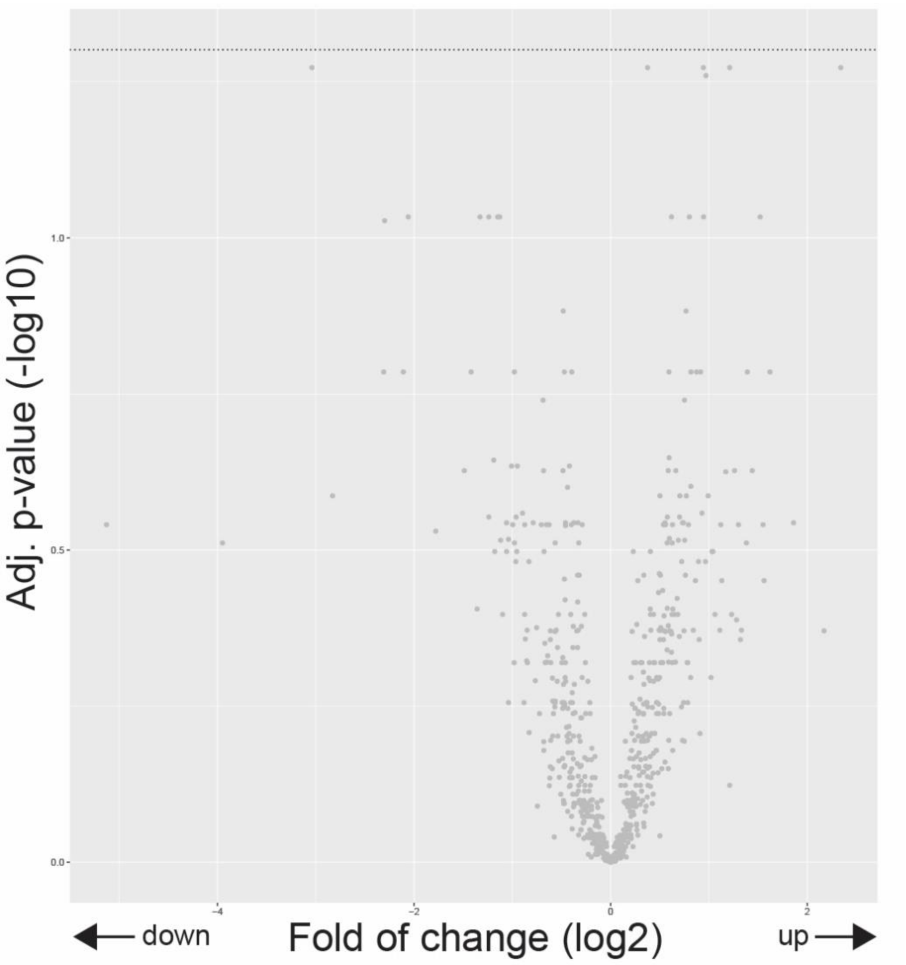

Supplementary Figure 2. Volcano plot showing over- and underexpressed genes in CCLR. Each dot represents a gene of the nCounter® PanCancer IO 360 Gene Expression Panel. Y-axis: p-values after the Benjamini-Hochberg procedure (-log10 scale). X-axis: Fold of change is calculated with CCNR as baseline reference (log2 ratio). Arrows indicate up- or downregulation. CCNR: Cervical cancer no recurrence; CCLR: Cervical cancer local recurrence.

Density of FoxP3+ cells in cervical cancer tissues and healthy controls

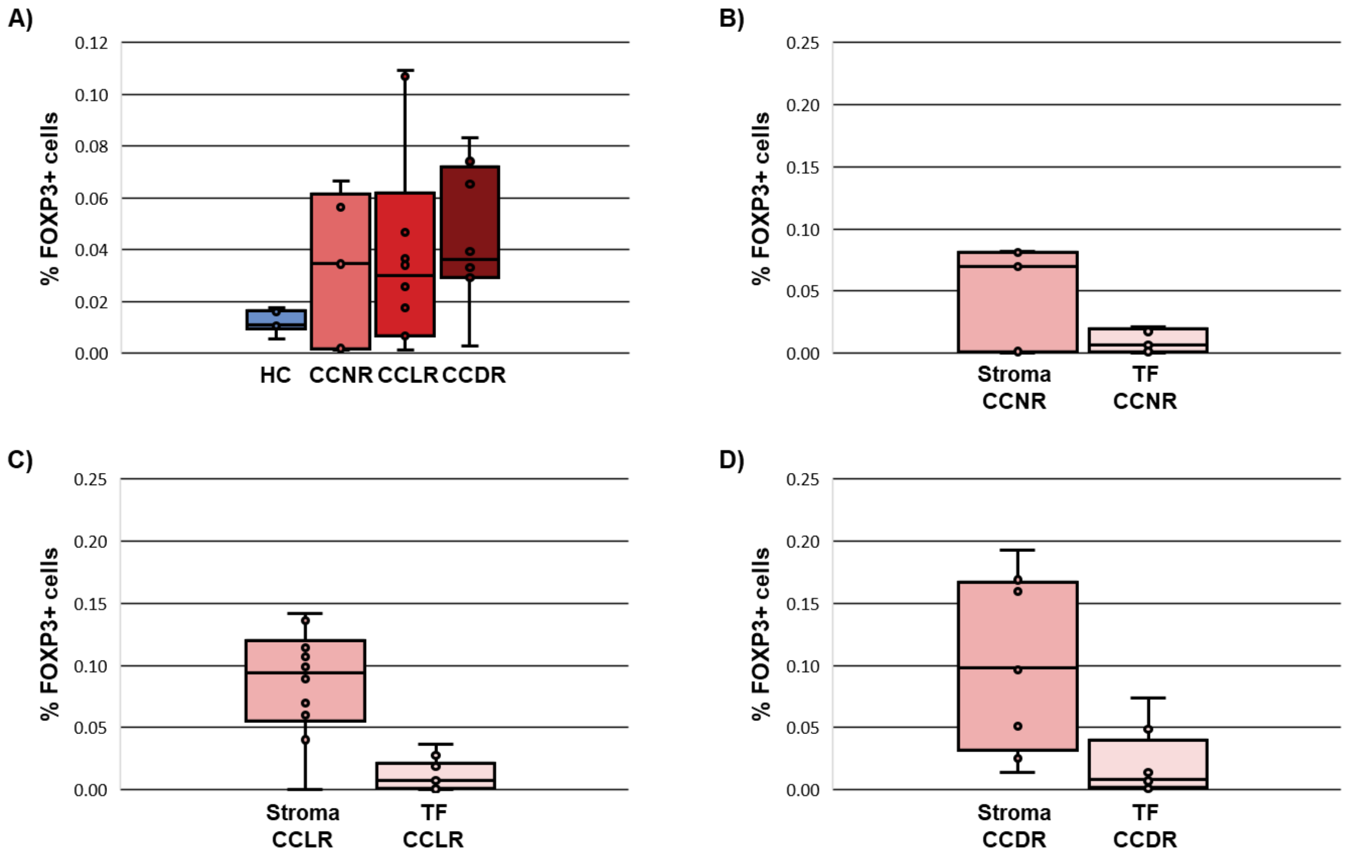

Supplementary Figure 3. The percentage of FoxP3+ cells is calculated according to surface FoxP3+ cells/surface of all cells *100. A) Density FoxP3+ cells in all groups. B) Density FoxP3+ cells in CCNR comparing stromal tissue and tumor fields. C) Density FoxP3+cells in CCLR comparing stromal tissue and tumor fields. D) Density FoxP3+ cells in CCDR comparing stromal tissue and tumor fields. TF: Tumor fields; HC: Healthy controls; CCLR: Cervical cancer local recurrence; CCDR: Cervical cancer distant recurrence; CCNR: Cervical cancer no recurrence

Expression of marker genes FoxP3 and IL2RA

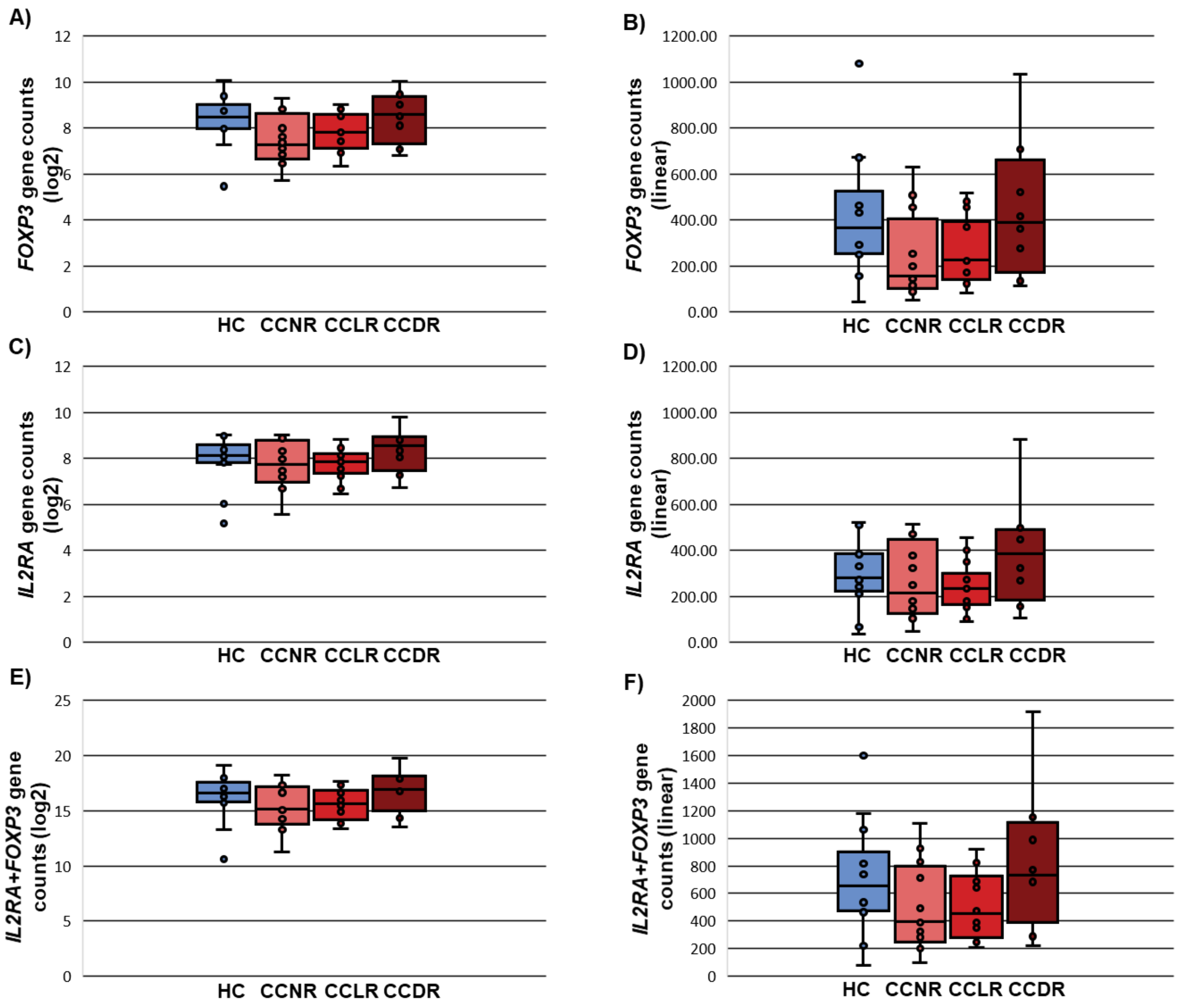

Supplementary Figure 4. The gene counts for FoxP3 and IL2RA are shown in both linear and log2 scales for all study groups. A) FoxP3 gene counts in log2-scale. B) FoxP3 gene counts in linear scale. C) IL2RA gene counts in log2-scale. D) IL2RA gene counts in a linear scale. E) Sum of FoxP3 and IL2RA gene counts in log2-scale. F) Sum of FoxP3 and IL2RA gene counts in linear scale. HC: Healthy controls; CCLR: Cervical cancer local recurrence; CCDR: Cervical cancer distant recurrence; CCNR: Cervical cancer no recurrence

Summary of the TCGA cervical cancer data

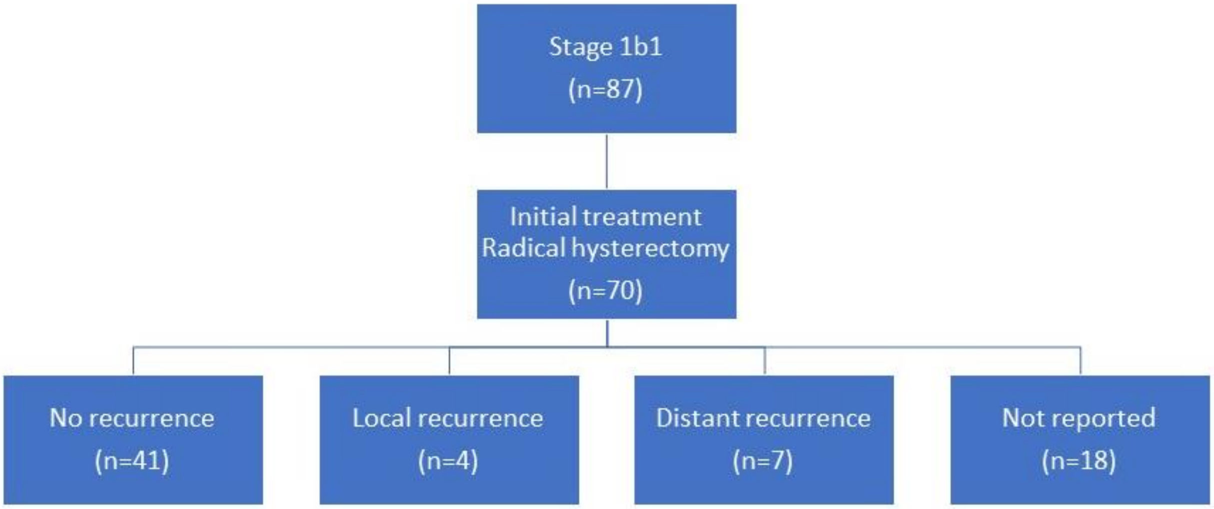

Supplementary Figure 5: The number of cervical cancer samples available in the TCGA data.
